# Association between physical activity and diabetes control: multiple cross-sectional studies and a prospective study in a population-based, Swiss cohort

**DOI:** 10.1136/bmjopen-2023-078929

**Published:** 2024-10-21

**Authors:** Gaël VonLanthen, Pedro Marques-Vidal

**Affiliations:** 1 Faculty of Biology and Medicine, University of Lausanne, Lausanne, Switzerland; 2 Medicine, Internal Medicine, Lausanne University Hospital (CHUV), Lausanne, Switzerland

**Keywords:** general diabetes, preventive medicine, epidemiologic studies

## Abstract

**Abstract:**

**Introduction:**

Physical activity (PA) is recommended in patients with type 2 diabetes mellitus (T2DM) to improve their glycaemic control. We aimed to assess PA levels among participants with controlled and uncontrolled T2DM.

**Research design and methods:**

Three cross-sectional analyses of a prospective cohort conducted in Lausanne, Switzerland. PA levels (sedentary, light, moderate and vigorous) were either self-reported via questionnaire (first and second survey) or objectively assessed using accelerometry (second and third survey). T2DM control was defined as glycaemia <7.0 mmol/L or glycated haemoglobin <6.5% (48 mmol/mol).

**Results:**

Data from 195 (30.3% women), 199 (30.1% women) and 151 (44.4% women) participants with T2DM were analysed in the first (2009–2012), second (2014–2017) and third (2018–2021) surveys. Approximately half of the participants did not have controlled glycaemia. Using subjective data, over 90% (first survey) and 75% (second survey) of participants reported moderate and vigorous PA >150 min/week. After multivariable adjustment, no differences were found regarding all types of self-reported PA levels between controlled and uncontrolled participants. Objective assessment of PA led to considerable differences according to the software used: 90% and 20% of participants with moderate and vigorous PA >150 min/week, respectively. After multivariable adjustment, no differences were found for all PA levels between controlled and uncontrolled participants, irrespective of the analytical procedure used. Using glycated haemoglobin, almost two-thirds of participants were considered as uncontrolled, and no differences were found for objectively assessed PA between controlled and uncontrolled participants.

**Conclusions:**

No differences in PA levels were found between participants with controlled and uncontrolled T2DM.

**STRENGTHS AND LIMITATIONS OF THIS STUDY**

- Physical activity levels were assessed using accelerometer.
- Diabetes control was assessed using both fasting plasma glucose and glycated haemoglobin.
- Study was conducted in a single location; hence, generalisability might be an issue.
- Possible selection bias exists as only motivated participants accepted to wear the accelerometer.

## Introduction

 Diabetes mellitus affects 537 million adults in the world, 90% of whom have type 2 diabetes mellitus (T2DM). It is predicted that this number of people with diabetes will increase to 783 million by 2045. Diabetes also has a financial cost estimated at US$966 billion, representing 9% of total adult health spending.^1^

Besides quitting smoking and adopting a healthy diet, physical activity (PA) is recommended in all patients with T2DM to improve their glycaemic control, insulin action, lipid levels and blood pressure,^2^ thus reducing the risk of cardiovascular disease. Simple activity such as walking 30 min/day can promote weight loss and improve glycaemic control.^3^ More structured exercise programmes are more effective to reduce insulin resistance in T2DM.^4^ Exercise programmes can be focused on aerobic, resistance or combined training, leading to significant improvements in haemoglobin A1c (HbA1c) levels.^5^ Still, it has been reported that patients with T2DM seldom adhere to the recommended amounts of PA. Indeed, barriers such as old age, female sex, lack of motivation, feeling of obligation, depression and fatigue can affect adhesion to recommended PA.^6^ For instance, in the EUROASPIRE IV and V studies, over half of patients with cardiovascular disease (CVD) and self-reported diabetes did not intend to do regular planned PA, and only one-quarter (26%) did.^7^

In Switzerland, it was estimated that, in 2021, 389 600 people aged between 20 and 79 years lived with diabetes, and 1 249 700 were affected by impaired glucose tolerance, with health costs amounting to US$4.9 billion.^8^ Still, the level of PA among people with T2DM and its impact on T2DM control have never been assessed.

Hence, we aimed to assess the effect of subjectively and objectively measured PA levels in subjects treated for T2DM according to diabetes control, using data from a population-based study.

## Materials and methods

### Participants

The CoLaus|PsyCoLaus study is a population-based prospective study assessing the clinical, biological and genetic determinants of CVD aged 35–75 years at baseline, living in the city of Lausanne, Switzerland.^9^ In each survey, participants answered questionnaires, underwent a clinical examination and blood samples were drawn for analyses. Recruitment began in June 2003 and ended in May 2006. The first follow-up was performed between April 2009 and September 2012; the second follow-up was performed between May 2014 and April 2017 and the third follow-up was performed between April 2018 and May 2021. For more details, see www.colaus-psycolaus.ch.

### Self-reported physical activity

Subjective PA was assessed using the Physical Activity Frequency Questionnaire (PAFQ). This self-reported questionnaire has been validated in the population of Geneva, Switzerland, and assesses the type and duration of 70 kinds of (non)professional activities and sports during the previous week. Sedentary status was defined as spending >90% of daily energy in activities below moderate intensity and high intensity (defined as requiring at least four times the basal metabolic rate (BMR)).^10^ BMR multiples are close to metabolic equivalent of task (MET) multiples, although MET multiples do not consider participant sex, age or height.

For the purpose of this study, each type of activity was categorised into sedentary behaviour (SB, <2 METs), light PA (LPA, 2 to <3 METs), moderate PA (MPA, 3–6 METs) and vigorous PA (VPA, >6 METs) according to the compendium of physical activities.^11^ Total PA was defined as the sum of LPA, MPA and VPA. For each item of the PAFQ, the time spent per week was computed as average hours per day multiplied by the number of days when the activity was performed. For each item category (ie, corresponding to SB, LPA, MPA or VPA), the times were summed up and divided by 7 to estimate an average daily time.

We chose to include SB in the analysis as we have previously shown that it is associated with an increased risk of developing T2DM.^12^

### Accelerometry-assessed physical activity

PA was objectively assessed using a wrist-worn triaxial accelerometer (GENEActiv, Activinsights, UK, www.activinsights.com). These devices are the same that have been used in the UK Biobank study,^13^ weigh 16 g and allow continuous monitoring of PA for a maximum of 45 days. The devices were preprogrammed with a 50 Hz sampling frequency and subsequently attached to the participants’ right wrist. Participants were requested to wear the device continuously for 14 days in their free-living conditions.

Raw accelerometry data were downloaded using the GENEActiv software V.2.9 (GENEActiv, Activinsights) and transformed into 1 min epoch files. Data were analysed using the GENEActiv Excel macro file ‘general PA’ V.1.9, which had been previously validated.^14^ A valid day was defined as ≥10 hours (ie, 600 min epoch) of diurnal wear time on weekdays and ≥8 hours (ie, 480 min epoch) on weekend days. The Excel macro file can be provided on request.

A second analysis was performed on the raw accelerometry data using the R-package GGIR V.1.5-9 (http://cran.r-project.org)^15^ with the thresholds defined by White *et al*,^16^ that is, an acceleration between 85 and 180 milli-g to define light PA, between 181 and 437 milli-g to define MPA and >437 milli-g to define VPA. The code used to analyse the data is provided in online supplemental file 1.

Participants were considered as complying with the recommendations if the weekly amount of MPA and VPA exceeded 150 min, as per European Society of Cardiology/European Association for the Study of Diabetes guidelines.^2^

### Diabetes assessment

Participants were asked whether they had been told they had diabetes and, if the answer was positive, if they were taking any medication (including insulin) to treat their diabetes. Participants were considered as presenting with treated diabetes if they reported taking any antidiabetic drug. Diabetes control was defined as a fasting plasma glucose <7 mmol/L; a second analysis was conducted using diabetes control defined as a glycated haemoglobin <6.5% (48 mmol/mol).

Blood was drawn in the fasting state and biological assays were performed by the Centre Hospitalier Universitaire Vaudois Clinical Laboratory on fresh blood samples within 2 hours of blood collection. The following analytical procedures (with maximum interbatch and intrabatch coefficients of variation) were used: glucose was measured by hexokinase method (1.6%–0.8%). In the second and third follow-ups, glycated haemoglobin levels were also measured by high performance liquid chromatography with the Bio-Rad, D-10 system, which has a measurement range of 3.8% (18 mmol/mol) to 18.5% (179 mmol/mol).

### Eligibility and exclusion criteria

All participants receiving treatment for diabetes were eligible for the study. Participants were excluded if they lacked PA data.

### Covariates

Participants were queried regarding their personal and family history of cardiovascular risk factors, medical treatment and socio-economic status. Educational level was categorised into low (mandatory or apprenticeship), medium (high school) and high (university). Smoking status was categorised into never, former and current.

Body weight and height were measured with participants barefoot and in light indoor clothes. Body weight was measured in kilograms to the nearest 100 g using a Seca scale (Hamburg, Germany). Height was measured to the nearest 5 mm using a Seca height gauge. Body mass index (BMI) was computed and categorised into normal (<25 kg/m^2^), overweight (≥25 and <30 kg/m^2^) and obese (≥30 kg/m^2^).

Blood pressure was measured using an Omron HEM-907 automated oscillometric sphygmomanometer after at least a 10 min rest in a seated position, and the average of the last two measurements was used. Hypertension was defined as a systolic blood pressure of ≥140 mm Hg, a diastolic blood pressure of ≥90 mm Hg or the presence of antihypertensive medication.

### Patient and public involvement

It was not possible to involve patients or the public in the design, or conduct, or reporting, or dissemination plans of our research.

### Statistical analysis

Statistical analyses were performed separately for each study period using Stata V.18.0 for windows (StataCorp, College Station, Texas, USA). Descriptive results were expressed as number of participants (percentage) for categorical variables and as average SD or median (IQR) for continuous variables. Bivariate analyses were performed using χ^2^ test or Fisher’s exact test for categorical variables and Student’s t-test, analysis of variance (ANOVA) or Kruskal-Wallis non-parametric test for continuous variables. Multivariable analysis of continuous data was performed using ANOVA and results were expressed as adjusted mean±SEM. Multivariable analysis of categorical data was performed using logistic regression and results were expressed as OR (95% CI). Multivariable analyses were conducted adjusting for sex (male, female), age (continuous), BMI categories (normal, overweight, obese), smoking status (never, former, current), educational level (low, medium, high).

A sensitivity analysis was conducted using multivariable linear regression adjusting for the same covariates to assess the association between PA and fasting plasma glucose or glycated haemoglobin. Results were expressed as standardised beta-coefficients.

Statistical significance was assessed for a two-sided test with p<0.05.

## Results

### Characteristics of participants

**Figure 1:**
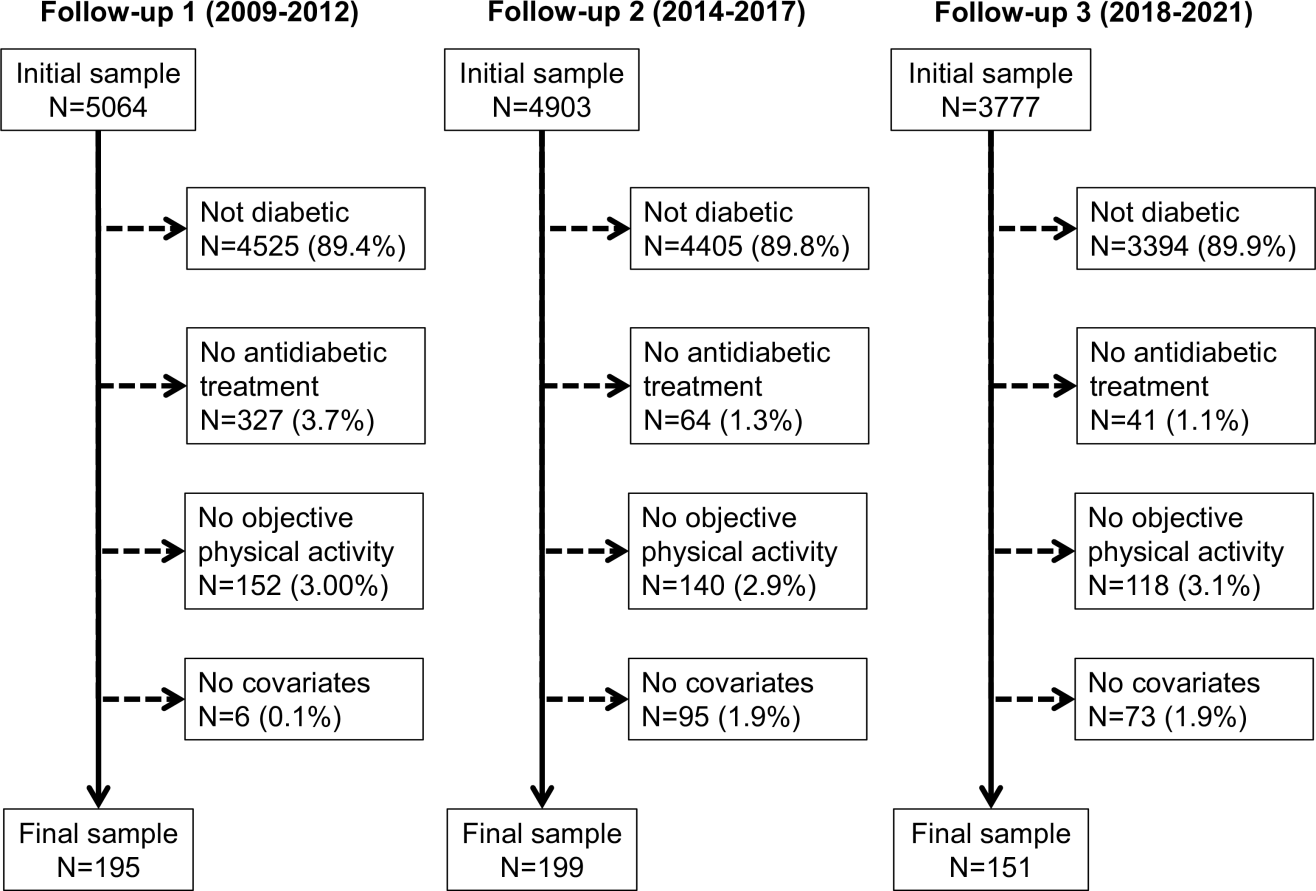
Selection procedure for the first, second and third follow-ups, CoLaus|PsyColaus study, Lausanne, Switzerland.

The selection procedure of the participants for the first, second and third follow-ups is summarised in figure 1 and the characteristics of the participants according to adequate or inadequate control of diabetes stratified by survey are provided in online supplemental table 1. Overall, one half of the participants treated for diabetes did not achieve adequate control. There were no consistent differences between controlled and uncontrolled participants, except that in the second follow-up, controlled participants were older and more frequently smokers.

### Sedentary behaviour and physical activity levels according to diabetes control as per fasting plasma glucose

The bivariate analysis of reported PA levels between controlled and uncontrolled participants for the first and the second follow-ups are presented in online supplemental table 2. Overall, over 90% and 75% of participants were compliant with the 150 min/week of MPA+VPA in the first and second surveys, respectively. Participants spent half of their time in SB and very little in VPA. No differences in PA (in absolute time or as percentage of day) were found between controlled and uncontrolled participants, and similar findings were obtained after multivariable adjustment (table 1).

**Table 1:** Multivariable analysis of self-reported PA by control group, stratified by survey: CoLaus|PsyCoLaus study, Lausanne, Switzerland

	First survey (2009–2012)			Second survey (2014–2017)		
	Not controlled	Controlled	P value	Not controlled	Controlled	P value
Sample size	121	74		52	48	
Intensity of PA (min/day)						
Sedentary	527±15	542±19	0.543	525±25	556±26	0.395
Light	197±10	166±13	0.056	204±16	176±16	0.237
Moderate	186±11	191±15	0.819	181±17	185±18	0.864
Vigorous	32±8	46±10	0.250	43±10	26±11	0.262
At least 150 min MVPA per week	1 (ref)	NC		1 (ref)	0.85 (0.35–2.09)	0.731*
Intensity of PA (% of daily time)						
Sedentary	56±1.5	57.3±2	0.608	54.8±2.4	58.8±2.5	0.257
Light	20.9±1	17.7±1.3	0.057	21.4±1.6	18.8±1.7	0.275
Moderate	19.7±1.2	20.2±1.5	0.798	19.3±1.9	19.6±1.9	0.291
Vigorous	3.4±0.8	4.8±1	0.260	4.6±1	2.8±1.1	0.257

Results are expressed as mean±SEM for continuous variables and as OR and (95% CI) for categorical variables. Statistical analysis by analysis of variance for continuous variables and by logistic regression for categorical variables, adjusted for sex (male, female), age (continuous), BMI categories (normal, overweight, obese), smoking status (never, former, current), educational level (low, medium, high).

* N=76 as several variables were dropped due to collinearity.

BMIbody mass indexMVPAmoderate and vigorous physical activityNCnot computablePA, physical activity

The results of the bivariate analysis of the objectively assessed PA levels using the GENEActiv macro between controlled and uncontrolled participants for the second and third follow-ups are presented in online supplemental table 3. Overall, over 90% of participants were compliant with the 150 min/week of MPA+VPA. Participants spent three quarters of their time in SB; conversely, they spent almost 2 hours/day on MPA. In the second survey, uncontrolled participants had higher levels and percentages of LPA and MPA, and a lower level and percentage of SB. In the third survey, no difference was found between controlled and uncontrolled participants. After multivariable adjustment, no significant differences were observed (table 2).

**Table 2:** Multivariable analysis, objectively assessed PA by control group as defined using fasting plasma glucose, stratified by survey: CoLaus|PsyCoLaus study, Lausanne, Switzerland

	Second survey (2014–2017)			Third survey (2018–2021)		
	Not controlled	Controlled	P value	Not controlled	Controlled	P value
Sample size	97	102		79	72	
Intensity of PA (min/day)						
Sedentary	609±12	634±11	0.131	601±14	591±15	0.634
Light	97±4	86±4	0.028	92±4	83±4	0.138
Moderate	122±7	106±7	0.124	122±9	119±9	0.798
Vigorous	1±1	1±1	0.684	1±1	1±1	0.476
At least 150 min MVPA per week	1 (ref)	0.94 (0.23–3.77)	0.925	1 (ref)	1.45 (0.39–5.42)	0.576
Intensity of PA (% of daily time)						
Sedentary	73.8±1.1	77.1±1.1	0.035	74.3±1.2	74.7±1.3	0.812
Light	11.7±0.4	10.2±0.4	0.011	11.1±0.4	10.3±0.4	0.280
Moderate	14.4±0.8	12.5±0.8	0.105	14.5±1.1	14.7±1.1	0.897
Vigorous	0.2±0.1	0.1±0.1	0.679	0.1±0.1	0.2±0.1	0.232

Results are expressed as mean±SEM for continuous variables and as OR (95% CI) for categorical variables. Statistical analysis by analysis of variance for continuous variables and by logistic regression for categorical variables, adjusted for sex (male, female), age (continuous), BMI categories (normal, overweight, obese), smoking status (never, former, current), educational level (low, medium, high). PA data were assessed using the GENEActiv macro file ‘general physical activity’ V.1.9.

BMIbody mass indexMVPAmoderate and vigorous physical activityPA, physical activity

online supplemental table 4 shows the bivariate analysis between controlled and uncontrolled participants of follow-ups 2 and 3, for objectively assessed PA, using the R-package GGIR. Overall, less than 25% of participants were compliant with the 150 min/week of MPA+VPA. Participants spent approximately one-quarter of an hour per day in MPA, and 90% of their time in SB. In the second survey, uncontrolled participants had higher levels and percentages of LPA and MPA, and a lower level and percentage of SB. After multivariable adjustment, no significant differences were observed (online supplemental table 5).

The results of the sensitivity analysis using multivariable linear regression are provided in online supplemental tables 6–8. Besides a significant negative association between LPA and glucose levels in the second follow-up for PA assessed by the MACRO procedure, which was not confirmed in the third follow-up, no other association between PA levels and glucose levels was found.

### Sedentary behaviour and physical activity levels according to diabetes control as per glycated haemoglobin

The results of the bivariate analysis of the objectively assessed PA levels using the GENEActiv macro between controlled and uncontrolled participants for the second and the third follow-ups are presented in online supplemental table 9. Almost two-thirds of participants were considered as uncontrolled. No differences were found between controlled and uncontrolled participants in bivariate and multivariable analyses (table 3).

**Table 3:** Multivariable analysis, objectively assessed PA by control group as defined by glycated haemoglobin, stratified by survey: CoLaus|PsyCoLaus study, Lausanne, Switzerland

	Second survey (2014–2017)			Third survey (2018–2021)		
	Not controlled	Controlled	P value	Not controlled	Controlled	P value
Sample size	123	76		95	56	
Intensity of PA (min/day)						
Sedentary	613±10	636±13	0.172	599±13	592±17	0.736
Light	94±3	87±4	0.216	89±4	85±5	0.543
Moderate	118±6	106±8	0.253	123±8	118±10	0.725
Vigorous	1±1	1±1	0.978	1±1	1±1	0.445
At least 150 min MVPA per week	1 (ref)	1.54 (0.41–5.79)	0.525	1 (ref)	1.11 (0.29–4.33)	0.879
Intensity of PA (% of daily time)						
Sedentary	74.6±1.0	76.9±1.2	0.143	74.4±1.1	74.8±1.5	0.821
Light	11.3±0.3	10.4±0.4	0.108	10.8±0.4	10.5±0.5	0.798
Moderate	14.0±0.7	12.6±0.9	0.225	14.7±0.9	14.5±1.2	0.884
Vigorous	0.1±0.1	0.1±0.1	0.925	0.1±0.1	0.2±0.1	0.394

Results are expressed as mean±SEM for continuous variables and as OR (95% CI) for categorical variables. Statistical analysis by analysis of variance for continuous variables and by logistic regression for categorical variables, adjusted for sex (male, female), age (continuous), BMI categories (normal, overweight, obese), smoking status (never, former, current), educational level (low, medium, high). PA data assessed using the GENEActiv macro file ‘general physical activity’ V.1.9.

BMIbody mass indexMVPAmoderate and vigorous physical activityPA, physical activity

The results of the bivariate and multivariable analysis of the objectively assessed PA levels using the R-package GGIR between controlled and uncontrolled participants for the second and the third follow-ups are presented in online supplemental table 10 (bivariate) and online supplemental table 11 (multivariable). Almost two-thirds of participants were considered as uncontrolled. No differences were found between controlled and uncontrolled participants in bivariate and multivariable analyses.

The results of the sensitivity analysis using multivariable linear regression are provided in online supplemental tables 12 and 13. No significant association between PA levels and glycated haemoglobin was found.

## Discussion

Our results show that over half of participants treated for type 2 diabetes are not controlled. They also show that neither self-reported nor objectively assessed PA levels differ according to diabetes control.

### Characteristics of participants

Overall, participants with controlled T2DM represented less than half of the participants in each of the three follow-ups. These values are lower than those reported in most European countries.^17 18^ The reasons for such a low control are not easily identifiable: no differences were found between controlled and uncontrolled participants for almost all covariates analysed, and a previous study showed no differences in dietary intakes.^19^ Hence, the factors associated with T2DM control may be less effective healthcare or differences in PA levels, which will be detailed in the next section. Overall, our results indicate that over half of treated diabetics do not achieve adequate control in this Swiss population-based sample.

### Sedentary behaviour and physical activity levels according to diabetes control

PA is a cost-saving treatment.^20 21^ Patients with T2DM who regularly participate in aerobic exercise have a better control of their disease.^22^ According to Swiss and international guidelines, it is recommended to spend 150 min/week doing moderate-to-vigorous PA.^23^

In our study, participants with T2DM reported over 150 min/day of MPA. Our findings suggest that most participants with T2DM comply with PA recommendations, although a reporting bias cannot be excluded. Conversely, the results of the objectively assessed PA differed considerably according to the analytical method applied. According to the GENEActiv macro, almost all participants treated for T2DM were compliant with current PA recommendations, while according to the R-package GGIR this percentage was less than 25%. These differences between analytical methods have been reported previously^24^ and raise the importance of standardisation of PA accelerometry measurements.^25^

After multivariable adjustment, no differences were found between controlled and uncontrolled participants regarding all PA levels, either as absolute time or as % of day. Our findings agree with a study conducted in Poland, where no differences in both subjectively and objectively assessed PA levels were found between controlled and uncontrolled participants.^26^ Conversely, our findings do not replicate those of two other studies, which showed significant improvement in glycaemic control in participants with T2DM when regular PA was part of a healthy lifestyle.^27 28^ Possible explanations include the methods used to categorise participants. For instance, both studies used self-filled questionnaire to categorise participants into active and inactive, while ours used both subjective and objective PA assessment. It is likely that the relatively small sample size of our study led to a low statistical power, and we cannot exclude an indication bias, with participants with uncontrolled T2DM being recommended to exercise more frequently than those who are controlled.

PA levels differed considerably according to the methodology used. The differences between reported and objectively assessed PA are known,^29^ and the differences in PA levels according to the software used to process the accelerometry data have also been detected previously.^24^ Overall, our results indicate that the method to assess PA might considerably impact the associations between PA and cardiometabolic risk factors. Hence, care should be taken when comparing findings from studies that used different software to assess PA.

Female sex, older age, comorbidities such as obesity and depression, lack of motivation and social influence have been suggested to decrease adherence to PA.^30^ It would thus be useful to consider these barriers in subjects with T2DM when prescribing regular PA^6^ and consider routine activities as domestic chores to increase PA.^31^

### Implications for clinical practice

Overall, our results suggest that people with diabetes exhibit the same PA behaviour irrespective of their fasting glucose or HbA1c levels. As PA is part of the management of T2DM,^2^ more emphasis should be put by clinicians to motivate their patients to be more active, different types of PA being effective.^5^ Still, doctors might not have either the time or the knowledge^32^ to adequately advise their patients regarding PA. Hence, postgraduate training regarding PA prescription is advised.^33^

### Strengths and limitations

The major strengths of this study is the use of both subjectively and objectively assessed PA. It used two different software to analyse PA and two different criteria (fasting plasma glucose and glycated haemoglobin) to define T2DM. The results were replicated in two time points and a population-based sample was used.

This study also has some limitations. First, the study was conducted in a single location, and results might not be extrapolated to other settings, although similar findings were obtained elsewhere.^26^ Second, a possible selection bias might have occurred, as more motivated participants may accept to wear the accelerometer more easily. Hence, it is likely that the amounts of PA might be overestimated, but not the comparisons between controlled and uncontrolled participants. Third, the cross-sectional design of this study cannot address the question whether effective PA levels can efficiently help manage diabetes. Still, our results are similar to those reported elsewhere,^34^ and suggest that PA levels should be implemented among people with diabetes. Finally, the amounts of LPA, MPA and VPA differed considerably according to the analytical procedure applied. This issue has already been discussed^24^ and recommendations have been issued.^24 35^ Furthermore, the results between controlled and uncontrolled participants were identical irrespective of the analytical procedure applied.

## Conclusion

In this population-based study focusing on participants treated for T2DM, no differences were found between controlled and uncontrolled T2DM regarding self-reported or objectively assessed PA levels.

## supplementary material

10.1136/bmjopen-2023-078929 online supplemental file 1

10.1136/bmjopen-2023-078929 online supplemental file 2

## Data Availability

Data may be obtained from a third party and are not publicly available.
