# Supplementary material for: Association between physical activity and diabetes control: multiple cross-sectional studies and a prospective study in a population-based, Swiss cohort
Source: BMJ Open. 2024 Oct 21;14(10):e078929. doi: 10.1136/bmjopen-2023-078929 (PMC11499834; doi:10.1136/bmjopen-2023-078929)
Supplement: online supplemental file 1 [file bmjopen-14-10-s001.pdf]

```
##########################################################################
#Loading packages
# USING THRESHOLDS AS DEFINED BY White and al. PMID 27936024)
# CHECK GGIR VERSION, IF NECESSARY INSTALL VERSION 9
##########################################################################

#install.packages(c("devtools"))
#require(devtools)
#install_version("GGIR", version="1.5-9")
#install.packages(c ("GENEAread", "zoo", "bitops", "data.table", "mmap"))
ev <- lapply("GGIR", library, character.only=T)
ev
search() # check if all is loaded



mode=c(1,2,3,4,5)

# Path for the folder where bin files are stored
datadir=  "F:/DATA/Input"

#pathname to folder where output should be written to "
# Changed 17.08.2017 to have ndayswindow=14 and maxdur=0

outputdir="F:/DATA/Output"
studyname="COLAUS"
f0 = 1
f1 = 1  # f1 = c() for all
g.shell.GGIR  (#-------------------------------
        # General parameters
        #-------------------------------
        do.parallel = FALSE,
```

```
 mode=mode,

datadir=datadir,

outputdir=outputdir,

studyname=studyname,

f0=f0,

f1=f1,

overwrite = TRUE,

do.imp=TRUE,

idloc=1,

print.filename=TRUE,

storefolderstructure = FALSE,

#-------------------------------

# Part 1 parameters:

#-------------------------------

windowsizes = c(5,900,3600),

#deziredtz="Europe/Bern",

do.cal=TRUE,

do.enmo = TRUE,

do.anglez=TRUE,

chunksize=1,

printsummary=TRUE,

#-------------------------------

# Part 2 parameters:

#-------------------------------

strategy = 1,

ndayswindow=14,

hrs.del.start = 1,

hrs.del.end = 1,

maxdur = 15,

includedaycrit = 16,

L5M5window = c(0,24),
```

```
M5L5res = 10,
winhr = c(5),
qlevels = c(c(1380/1440),c(1410/1440)),
qwindow=c(0,24),
ilevels = c(seq(0,400,by=50),8000),
mvpathreshold =c(182),
#-------------------------------
# Part 3 parameters:
#-------------------------------
timethreshold= c(5),
anglethreshold=5,
ignorenonwear = TRUE,
#-------------------------------
# Part 4 parameters:
#-------------------------------
excludefirstlast = FALSE,
includenightcrit = 16,
def.noc.sleep = c(21,9),
#loglocation= "pathname to a sleeplog - if using one",
outliers.only = FALSE,
criterror = 4,
relyonsleeplog = FALSE,
sleeplogidnum = TRUE,
colid=1,
coln1=2,
do.visual = FALSE,
nnights = 14,
#-------------------------------
# Part 5 parameters:
#-------------------------------
# Key functions: Merging physical activity with sleep analyses
```

```
#threshold.lig = c(30,40,50),
#threshold.mod = c(100,120),
#threshold.vig = c(400,500),
threshold.lig = c(85),
threshold.mod = c(181),
threshold.vig = c(437),
boutcriter = 0.8,
boutcriter.in = 0.9,
boutcriter.lig = 0.8,
boutcriter.mvpa = 0.8,
#boutdur.in = c(10,20,30),
#boutdur.lig = c(1,5,10),
#boutdur.mvpa = c(1,5,10),
boutdur.in = c(10),
boutdur.lig = c(10),
boutdur.mvpa = c(10),
timewindow = c("WW"),
#-------------------------------
# Report generation
#-------------------------------
do.report=c(5))
```
