# Supplementary material for: Association between physical activity and diabetes control: multiple cross-sectional studies and a prospective study in a population-based, Swiss cohort
Source: BMJ Open. 2024 Oct 21;14(10):e078929. doi: 10.1136/bmjopen-2023-078929 (PMC11499834; doi:10.1136/bmjopen-2023-078929)
Supplement: online supplemental file 2 [file bmjopen-14-10-s002.pdf]

Supplementary information

Table 1: characteristics of participants according to diabetes control as per fasting plasma glucose, stratified by survey, CoLaus|PsyColaus study, Lausanne, Switzerland.

	First survey (2009-2012)			Second survey (2014-2017)			Third survey (2018-2021)		
	**Not controlled**	**Controlled**	**p-value**	**Not controlled**	**Controlled**	**p-value**	**Not controlled**	**Controlled**	**p-value**
Sample size	121	74		97	102		79	72	
Women (%)	37 (30.6)	22 (29.7)	1.000	28 (28.9)	32 (31.4)	0.758	32 (40.5)	35 (48.6)	0.330
Age (years)	65.1 ± 8.8	65.9 ± 8.4	0.519	66.6 ± 8.7	69.5 ± 8.8	0.020	68.8 ± 8.7	68.1 ± 8.7	0.652
Swiss born (%)	85 (70.3)	43 (58.1)	0.090	59 (60.8)	67 (65.7)	0.556	55 (69.6)	42 (58.3)	0.175
Education (%)			0.212			0.589			1.000
High	11 (9.1)	13 (17.6)		12 (12.4)	12 (11.9)		9 (11.4)	8 (11.1)	
Middle	29 (24.0)	18 (24.3)		24 (24.7)	19 (18.8)		18 (22.8)	17 (23.6)	
Low	81 (66.9)	43 (58.1)		61 (62.9)	70 (69.3)		52 (65.8)	47 (65.3)	
BMI (kg/m^2)	30.5 ± 5.6	29.4 ± 4.1	0.161	30.5 ± 4.9	30.6 ± 5.1	0.924	29.8 ± 4.6	29.0 ± 5.6	0.378
BMI categories (%)			0.711			1.000			0.319
Normal	17 (14.1)	10 (13.5)		11 (11.5)	12 (11.8)		11 (13.9)	15 (20.8)	
Overweight	50 (41.3)	35 (47.3)		37 (38.5)	39 (38.2)		33 (41.8)	33 (45.8)	
Obese	54 (44.6)	29 (39.2)		48 (50.0)	51 (50.0)		35 (44.3)	24 (33.3)	
Smoking categories (%)			0.770			0.022			0.950
Never	37 (30.6)	25 (33.8)		26 (26.8)	39 (38.2)		29 (36.7)	28 (38.9)	
Former	65 (53.7)	40 (54.1)		57 (58.8)	40 (39.2)		38 (48.1)	34 (47.2)	
Current	19 (15.7)	9 (12.2)		14 (14.4)	23 (22.6)		12 (15.2)	10 (13.9)	
Hypertension (%)	99 (81.8)	55 (74.3)	0.277	76 (78.4)	80 (78.4)	1.000	67 (84.8)	57 (79.2)	0.401
Hypolipidemic ttt (%)	67 (55.4)	51 (68.9)	0.071						
History of CVD (%)	18 (14.9)	12 (16.2)	0.839	18 (18.6)	20 (19.6)	0.859	16 (20.3)	13 (18.1)	0.837

BMI, body mass index; CVD, cardiovascular disease; ttt, treatment. Results expressed as mean ± standard deviation for continuous variables or as number of participants (percentage) for categorical variables. Statistical analysis by student's t-test or chi-square test.

Supplementary table 2: bivariate analysis, self-reported physical activity by diabetes control group as defined by fasting plasma glucose, stratified by survey, CoLaus|PsyCoLaus study, Lausanne, Switzerland.

	**First survey (2009-2012)**			**Second survey (2014-2017)**		
	**Not controlled**	**Controlled**	**p-value**	**Not controlled**	**Controlled**	**p-value**
Sample size	121	74		52	48	
Intensity of PA (min/day)						
Sedentary	522 [394 - 660]	557 [449 - 651]	0.379	555 [405 - 665]	532 [469 - 648]	0.637
Light	185 [107 - 272]	156 [109 - 243]	0.128	168 [125 - 249]	157 [123 - 242]	0.567
Moderate	164 [95 - 249]	155 [107 - 265]	0.862	143 [105 - 246]	161 [104 - 221]	0.992
Vigorous	0.7 [0 - 4]	0.8 [0 - 4.5]	0.995	18 [0 - 46]	15 [0 - 55]	0.997
At least 150 minutes MVPA per week	118 (97.5)	73 (98.7)	§	51 (78.5)	45 (76.3)	0.270
Intensity of PA (% of daily time)						
Sedentary	57.2 [42.4 - 68.8]	59.4 [46.8 - 68.4]	0.418	60.1 [44.6 - 68.0]	58.0 [48.3 - 68.0]	0.551
Light	19.3 [11.2 - 29.7]	16.6 [12.0 - 24.5]	0.108	18.5 [13.8 - 26.7]	17.7 [12.8 - 26.6]	0.801
Moderate	17.2 [10.7 - 25.8]	16.2 [11.2 - 26.9]	0.823	15.0 [10.3 - 26.8]	16.7 [11.4 - 24.2]	0.866
Vigorous	6.0 [0 - 37.0]	6.0 [0 - 39.0]	0.980	1.9 [0 - 5.2]	1.4 [0 - 6.0]	0.963

PA, physical activity; MVPA, moderate and vigorous physical activity. Results expressed as median [interquartile range] for continuous variables and as number of participants (column percentage) for categorical variables. Statistical analysis by Kruskal-Wallis test for continuous variables and chi-square or Fisher's exact test (§) for categorical variables.

Supplementary table 3: bivariate analysis, objectively assessed physical activity by diabetes control group as defined by fasting plasma glucose, stratified by survey, CoLaus|PsyCoLaus study, Lausanne, Switzerland.

	Second survey (2014-2017)			Third survey (2018-2021)		
	**Not controlled**	**Controlled**	**p-value**	**Not controlled**	**Controlled**	**p-value**
Sample size	97	102		79	72	
Intensity of PA (min/day)						
Sedentary	624 [522 - 691]	640 [561 - 734]	0.061	625 [515 - 693]	588 [503 - 690]	0.341
Light	92 [69 - 124]	83 [58 - 111]	0.024	87 [65 - 115]	83 [68 - 102]	0.378
Moderate	108 [65 - 165]	91 [54 - 134]	0.057	117 [56 - 161]	111 [60 - 160]	0.780
Vigorous	0 [0 - 1]	0 [0 - 1]	0.021	0 [0 - 2]	0 [0 - 2]	0.683
At least 150 minutes MVPA per week (%)	93 (95.9)	94 (92.2)	0.271	71 (89.9)	67 (93.1)	0.486
Intensity of PA (% of daily time)						
Sedentary	74.7 [67.0 - 82.6]	79.2 [72.1 - 85.1]	0.011	75.7 [68.1 - 84.9]	74.5 [66.7 - 82.3]	0.587
Light	11.1 [9.1 - 14.1]	9.8 [7.5 - 13.0]	0.008	11 [8.0 - 13.1]	10.7 [8.4 - 12.9]	0.631
Moderate	12.5 [8.3 - 20.0]	10.3 [7.3 - 16.4]	0.039	14.1 [6.9 - 18.9]	14.0 [8.4 - 18.9]	0.479
Vigorous	0.1 [0 - 0.2]	0 [0 - 0.1]	0.021	0 [0 - 0.2]	0 [0 - 0.2]	0.582

PA, physical activity; MVPA, moderate and vigorous physical activity. Results expressed as median [interquartile range] for continuous variables and as number of participants (column percentage) for categorical variables. Statistical analysis by Kruskal-Wallis test for continuous variables and chi-square or Fisher's exact test (§) for categorical variables. Physical activity data assessed using the GENEActiv macro file 'General physical activity' version 1.9.

Supplementary table 4: bivariate analysis, objectively assessed physical activity by diabetes control group as defined by fasting plasma glucose, stratified by survey, CoLaus|PsyCoLaus study, Lausanne, Switzerland.

	**Second survey (2014-2017)**			**Third survey (2018-2021)**		
	**Not controlled**	**Controlled**	**p-value**	**Not controlled**	**Controlled**	**p-value**
Sample size	95	100		31	27	
Intensity of PA (min/day)						
Sedentary	750 [708 - 811]	770 [722 - 822]	0.123	768 [725 - 809]	742 [706 - 821]	0.591
Light	72 [43 - 107]	60 [35 - 89]	0.076	81 [43 - 106]	66 [40 - 105]	0.198
Moderate	11 [6 - 22]	9 [3 - 16]	0.039	13 [6 - 22]	9 [6 - 19]	0.239
Vigorous	1 [0 - 1]	0 [0 - 1]	0.041	1 [0 - 2]	0 [0 - 1]	0.080
At least 150 minutes MVPA per week	19 (20.0)	13 (13.0)	0.187	8 (25.8)	6 (22.2)	0.750
Intensity of PA (% of daily time)						
Sedentary	90.2 [85 - 94.1]	92.1 [87.8 - 95.1]	0.057	89.1 [85.5 - 94.0]	91.6 [86.6 - 95.0]	0.233
Light	8.2 [5.3 - 12.2]	6.8 [4.3 - 10.5]	0.062	9.1 [5.4 - 12.3]	7.3 [4.4 - 11.1]	0.252
Moderate	1.3 [0.7 - 2.4]	1.1 [0.4 - 1.8]	0.032	1.6 [0.7 - 2.4]	1.0 [0.7 - 2.2]	0.239
Vigorous	0.1 [0 - 0.2]	0 [0 - 0.1]	0.033	0.1 [0 - 0.2]	0 [0 - 0.1]	0.072

PA, physical activity; MVPA, moderate and vigorous physical activity. Results expressed as median [interquartile range] for continuous variables and as number of participants (column percentage) for categorical variables. Statistical analysis by Kruskal-Wallis test for continuous variables and chi-square for categorical variables. Physical activity data assessed using the R-package GGIR version 1.5–9.

Supplementary table 5: multivariable analysis, objectively assessed physical activity by control group as defined by fasting plasma glucose, stratified by survey, CoLaus|PsyCoLaus study, Lausanne, Switzerland.

	Second survey (2014-2017)			Third survey (2018-2021)		
	**Not controlled**	**Controlled**	**p-value**	**Not controlled**	**Controlled**	**p-value**
Sample size	95	100		31	27	
Intensity of PA (min/day)						
Sedentary	761 ± 7	769 ± 7	0.403	756 ± 14	770 ± 15	0.527
Light	78 ± 4	69 ± 4	0.184	85 ± 9	71 ± 9	0.317
Moderate	15 ± 1	13 ± 1	0.509	18 ± 3	15 ± 3	0.528
Vigorous	1 ± 1	1 ± 1	0.394	1 ± 1	1 ± 1	0.370
At least 150 minutes MVPA per week	1 (ref)	0.85 (0.34 - 2.14)	0.727	1 (ref)	0.39 (0.07 - 2.07)	0.268
Intensity of PA (% of daily time)						
Sedentary	89.3 ± 0.6	90.4 ± 0.6	0.212	88.2 ± 1.3	90.0 ± 1.3	0.353
Light	8.9 ± 0.5	8.0 ± 0.5	0.177	9.6 ± 0.9	8.2 ± 1.0	0.316
Moderate	1.7 ± 0.1	1.5 ± 0.1	0.501	2.0 ± 0.3	1.7 ± 0.4	0.581
Vigorous	0.2 ± 0.1	0.1 ± 0.1	0.432	0.2 ± 0.1	0.1 ± 0.1	0.395

PA, physical activity; MVPA, moderate and vigorous physical activity. Results are expressed as mean ± sem for continuous variables and as odds ratio and (95% confidence interval) for categorical variables. Statistical analysis by analysis of variance for continuous variables and by logistic regression for categorical variables, adjusted for sex (male, female), age (continuous), BMI categories (normal, overweight, obese), smoking status (never, former, current), educational level (low, medium, high). Physical activity data assessed using the R-package GGIR version 1.5–9.

Table 1: multivariable analysis, self-reported physical activity by control group, stratified by survey, CoLaus|PsyCoLaus study, Lausanne, Switzerland.

	First survey (2009-2012)			Second survey (2014-2017)		
	**Not controlled**	**Controlled**	**p-value**	**Not controlled**	**Controlled**	**p-value**
Sample size	121	74		52	48	
Intensity of PA (min/day)						
Sedentary	527 ± 15	542 ± 19	0.543	525 ± 25	556 ± 26	0.395
Light	197 ± 10	166 ± 13	0.056	204 ± 16	176 ± 16	0.237
Moderate	186 ± 11	191 ± 15	0.819	181 ± 17	185 ± 18	0.864
Vigorous	32 ± 8	46 ± 10	0.250	43 ± 10	26 ± 11	0.262
At least 150 minutes MVPA per week	1 (ref)	NC		1 (ref)	0.85 (0.35 - 2.09)	0.731 §
Intensity of PA (% of daily time)						
Sedentary	56 ± 1.5	57.3 ± 2	0.608	54.8 ± 2.4	58.8 ± 2.5	0.257
Light	20.9 ± 1	17.7 ± 1.3	0.057	21.4 ± 1.6	18.8 ± 1.7	0.275
Moderate	19.7 ± 1.2	20.2 ± 1.5	0.798	19.3 ± 1.9	19.6 ± 1.9	0.291
Vigorous	3.4 ± 0.8	4.8 ± 1	0.260	4.6 ± 1	2.8 ± 1.1	0.257

PA, physical activity; MVPA, moderate and vigorous physical activity Results are expressed as standardized beta coefficients. Statistical analysis by linear regression adjusted for sex (male, female), age (continuous), BMI categories (normal, overweight, obese), smoking status (never, former, current), educational level (low, medium, high).

Supplementary table 6: multivariable analysis, association between self-reported physical activity and fasting plasma glucose, stratified by survey, CoLaus|PsyCoLaus study, Lausanne, Switzerland.

	**First survey (2009-2012)**	**p-value**	**Second survey (2014-2017)**	**p-value**
Sample size	195		100	
Intensity of PA (min/day)				
Sedentary	0.040	0.599	-0.080	0.455
Light	0.068	0.378	0.074	0.493
Moderate	0.003	0.968	0.070	0.517
Vigorous	-0.139	0.059	0.051	0.635
Intensity of PA (% of daily time)				
Sedentary	0.028	0.716	-0.120	0.271
Light	0.066	0.388	0.055	0.613
Moderate	0.003	0.972	0.083	0.439
Vigorous	-0.141	0.055	0.041	0.700

PA, physical activity; MVPA, moderate and vigorous physical activity. Results are expressed as standardized beta coefficients. Statistical analysis by linear regression adjusted for sex (male, female), age (continuous), BMI categories (normal, overweight, obese), smoking status (never, former, current), educational level (low, medium, high).

Supplementary table 7: multivariable analysis, association between objectively assessed physical activity and fasting plasma glucose, stratified by survey, CoLaus|PsyCoLaus study, Lausanne, Switzerland.

	Second survey (2014-2017)	p-value	Third survey (2018-2021)	p-value
Sample size	199		151	
Intensity of PA (min/day)				
Sedentary	-0.072	0.340	0.081	0.360
Light	0.203	0.005	0.126	0.152
Moderate	0.140	0.069	-0.051	0.589
Vigorous	0.012	0.871	-0.019	0.831
Intensity of PA (% of daily time)				
Sedentary	-0.145	0.061	0.021	0.827
Light	0.187	0.011	0.104	0.242
Moderate	0.103	0.188	-0.072	0.449
Vigorous	-0.007	0.928	-0.029	0.742

PA, physical activity; MVPA, moderate and vigorous physical activity. Results are expressed as standardized beta coefficients. Statistical analysis by linear regression adjusted for sex (male, female), age (continuous), BMI categories (normal, overweight, obese), smoking status (never, former, current), educational level (low, medium, high). Physical activity data assessed using the GENEActiv macro file 'General physical activity' version 1.9.

Supplementary table 8: multivariable analysis, association between objectively assessed physical activity and fasting plasma glucose, stratified by survey, CoLaus|PsyCoLaus study, Lausanne, Switzerland.

	**Second survey (2014-2017)**	**p-value**	**Third survey (2018-2021)**	**p-value**
Sample size	195		58	
Intensity of PA (min/day)				
Sedentary	-0.071	0.352	0.121	0.433
Light	0.154	0.052	-0.105	0.532
Moderate	0.078	0.339	-0.099	0.562
Vigorous	0.021	0.782	0.055	0.726
Intensity of PA (% of daily time)				
Sedentary	-0.141	0.080	0.256	0.102
Light	0.154	0.053	-0.101	0.547
Moderate	0.074	0.372	-0.100	0.557
Vigorous	0.012	0.880	0.067	0.670

PA, physical activity; MVPA, moderate and vigorous physical activity. Results are expressed as standardized beta coefficients. Statistical analysis by linear regression adjusted for sex (male, female), age (continuous), BMI categories (normal, overweight, obese), smoking status (never, former, current), educational level (low, medium, high). Physical activity data assessed using the R-package GGIR version 1.5–9.

Supplementary table 9: bivariate analysis, objectively assessed physical activity by diabetes control group as defined by glycated haemoglobin, stratified by survey, CoLaus|PsyCoLaus study, Lausanne, Switzerland.

	**Second survey (2014-2017)**			**Third survey (2018-2021)**		
	**Not controlled**	**Controlled**	**p-value**	**Not controlled**	**Controlled**	**p-value**
Sample size	123	76		95	56	
Intensity of PA (min/day)						
Sedentary	626 [532 - 697]	638 [569 - 731]	0.151	600 [509 - 697]	603 [520 - 687]	0.742
Light	89 [65 - 121]	81 [59 - 110]	0.113	90 [63 - 112]	82 [70 - 100]	0.455
Moderate	106 [62 - 162]	89 [54 - 134]	0.140	123 [63 - 166]	102 [53 - 151]	0.552
Vigorous	0 [0 - 1]	0 [0 - 1]	0.139	0 [0 - 2]	0 [0 - 2]	0.843
At least 150 minutes MVPA per week (%)	116 (94.3)	71 (93.4)	0.798	87 (91.6)	51 (91.1)	0.914
Intensity of PA (% of daily time)						
Sedentary	75.0 [68.2 - 83.1]	79.0 [72.0 - 85.2]	0.082	73.9 [66.3 - 83.9]	75.5 [68.4 - 82.3]	0.726
Light	11.0 [8.9 - 13.7]	9.9 [7.1 - 13.2]	0.074	11.2 [8.0 - 13.2]	10.1 [8.5 - 12.6]	0.520
Moderate	12.5 [7.9 - 19.6]	10.3 [7.2 - 16.7]	0.106	14.6 [7.6 - 19.4]	12.3 [7.6 - 17.7]	0.540
Vigorous	0 [0 - 0.2]	0 [0 - 0.1]	0.144	0 [0 - 0.2]	0 [0 - 0.2]	0.777

PA, physical activity; MVPA, moderate and vigorous physical activity. Results expressed as median [interquartile range] for continuous variables and as number of participants (column percentage) for categorical variables. Statistical analysis by Kruskal-Wallis test for continuous variables and chi-square for categorical variables. Physical activity data assessed using the GENEActiv macro file 'General physical activity' version 1.9.

Supplementary table 10: bivariate analysis, objectively assessed physical activity by diabetes control group as defined by glycated haemoglobin, stratified by survey, CoLaus|PsyCoLaus study, Lausanne, Switzerland.

	**Second survey (2014-2017)**			**Third survey (2018-2021)**		
	**Not controlled**	**Controlled**	**p-value**	**Not controlled**	**Controlled**	**p-value**
Sample size	119	76		38	20	
Intensity of PA (min/day)						
Sedentary	752 [719 - 812]	770 [722 - 827]	0.210	764 [727 - 808]	742 [706 - 819]	0.612
Light	69 [40 - 105]	60 [35 - 85]	0.146	77 [41 - 106]	68 [45 - 97]	0.935
Moderate	10 [5 - 19]	9 [4 - 17]	0.344	13 [6 - 22]	10 [7 - 17]	0.731
Vigorous	1 [0 - 1]	0 [0 - 1]	0.317	1 [0 - 2]	0 [0 - 1]	0.185
At least 150 minutes MVPA per week (%)	20 (16.8)	12 (15.8)	0.852	10 (26.3)	4 (20.0)	0.593
Intensity of PA (% of daily time)						
Sedentary	90.4 [85.5 - 94.3]	92.1 [87.6 - 95.1]	0.172	89.6 [85.5 - 94.1]	91.3 [87.1 - 93.5]	0.806
Light	7.9 [5.1 - 12.1]	6.9 [4.3 - 10.1]	0.116	8.6 [5.0 - 12.3]	7.6 [5.4 - 11.0]	0.909
Moderate	1.2 [0.6 - 2.2]	1.1 [0.4 - 2.0]	0.324	1.5 [0.7 - 2.4]	1.1 [0.8 - 2.0]	0.659
Vigorous	0.1 [0 - 0.2]	0 [0 - 0.2]	0.113	0.1 [0 - 0.2]	0.1 [0 - 0.1]	0.191

PA, physical activity; MVPA, moderate and vigorous physical activity. Results expressed as median [interquartile range] for continuous variables and as number of participants (column percentage) for categorical variables. Statistical analysis by Kruskal-Wallis test for continuous variables and chi-square for categorical variables. Physical activity data assessed using the R-package GGIR version 1.5–9.

Supplementary table 11: multivariable analysis, objectively assessed physical activity by control group as defined by glycated haemoglobin, stratified by survey, CoLaus|PsyCoLaus study, Lausanne, Switzerland.

	Second survey (2014-2017)			Third survey (2018-2021)		
	**Not controlled**	**Controlled**	**p-value**	**Not controlled**	**Controlled**	**p-value**
Sample size	119	76		38	20	
Intensity of PA (min/day)						
Sedentary	761 ± 6	770 ± 8	0.353	766 ± 12	756 ± 17	0.664
Light	76 ± 4	68 ± 5	0.221	76 ± 8	84 ± 11	0.570
Moderate	13 ± 1	15 ± 1	0.494	16 ± 3	18 ± 4	0.614
Vigorous	1 ± 1	1 ± 1	0.974	1 ± 1	1 ± 1	0.238
At least 150 minutes MVPA per week	1 (ref)	1.31 (0.53 - 3.28)	0.560	1 (ref)	0.62 (0.11 - 3.42)	0.586
Intensity of PA (% of daily time)						
Sedentary	89.5 ± 0.5	90.3 ± 0.7	0.392	89.4 ± 1.1	88.3 ± 1.6	0.571
Light	8.8 ± 0.4	7.9 ± 0.5	0.199	8.6 ± 0.8	9.5 ± 1.2	0.554
Moderate	1.5 ± 0.1	1.7 ± 0.2	0.484	1.8 ± 0.3	2.1 ± 0.4	0.580
Vigorous	0.1 ± 0.1	0.1 ± 0.1	0.982	0.2 ± 0.1	0.1 ± 0.1	0.228

PA, physical activity; MVPA, moderate and vigorous physical activity. Results are expressed as mean ± sem for continuous variables and as odds ratio and (95% confidence interval) for categorical variables. Statistical analysis by analysis of variance for continuous variables and by logistic regression for categorical variables, adjusted for sex (male, female), age (continuous), BMI categories (normal, overweight, obese), smoking status (never, former, current), educational level (low, medium, high). Physical activity data assessed using the R-package GGIR version 1.5–9.

Supplementary table 12: multivariable analysis, association between objectively assessed physical activity and glycated haemoglobin, stratified by survey, CoLaus|PsyCoLaus study, Lausanne, Switzerland.

	**Second survey (2014-2017)**	**p-value**	**Third survey (2018-2021)**	**p-value**
Sample size	199		151	
Intensity of PA (min/day)				
Sedentary	-0.072	0.340	0.081	0.360
Light	0.203	0.005	0.126	0.152
Moderate	0.140	0.069	-0.051	0.589
Vigorous	0.012	0.871	-0.019	0.831
Intensity of PA (% of daily time)				
Sedentary	-0.145	0.061	0.021	0.827
Light	0.187	0.011	0.104	0.242
Moderate	0.103	0.188	-0.072	0.449
Vigorous	-0.007	0.928	-0.029	0.742

PA, physical activity; MVPA, moderate and vigorous physical activity. Results are expressed as standardized beta coefficients. Statistical analysis by linear regression adjusted for sex (male, female), age (continuous), BMI categories (normal, overweight, obese), smoking status (never, former, current), educational level (low, medium, high). Physical activity data assessed using the GENEActiv macro file 'General physical activity' version 1.9.

Supplementary table 13: multivariable analysis, association between objectively assessed physical activity and glycated haemoglobin, stratified by survey, CoLaus|PsyCoLaus study, Lausanne, Switzerland.

	**Second survey (2014-2017)**	**p-value**	**Third survey (2018-2021)**	**p-value**
Sample size	195		58	
Intensity of PA (min/day)				
Sedentary	-0.071	0.352	0.121	0.433
Light	0.154	0.052	-0.105	0.532
Moderate	0.078	0.339	-0.099	0.562
Vigorous	0.021	0.782	0.055	0.726
Intensity of PA (% of daily time)				
Sedentary	-0.141	0.080	0.256	0.102
Light	0.154	0.053	-0.101	0.547
Moderate	0.074	0.372	-0.100	0.557
Vigorous	0.012	0.880	0.067	0.670

PA, physical activity; MVPA, moderate and vigorous physical activity. Results are expressed as standardized beta coefficients. Statistical analysis by linear regression adjusted for sex (male, female), age (continuous), BMI categories (normal, overweight, obese), smoking status (never, former, current), educational level (low, medium, high). Physical activity data assessed using the R-package GGIR version 1.5–9.
