# Supplementary material for: Association between physical activity and diabetes control: multiple cross-sectional studies and a prospective study in a population-based, Swiss cohort
Source: BMJ Open. 2024 Oct 21;14(10):e078929. doi: 10.1136/bmjopen-2023-078929 (PMC11499834; doi:10.1136/bmjopen-2023-078929)
Supplement: Supplementary file 3 [file bmjopen-2023-078929.reviewer_comments.pdf]

PEER REVIEW HISTORY

BMJ Open publishes all reviews undertaken for accepted manuscripts. Reviewers are asked to complete a checklist review form and are provided with free text boxes to elaborate on their assessment. These free text comments are reproduced below.

ARTICLE DETAILS

Title (Provisional)

ASSOCIATION BETWEEN PHYSICAL ACTIVITY AND DIABETES CONTROL: MULTIPLE CROSS-SECTIONAL STUDIES AND A PROSPECTIVE STUDY IN A POPULATION-BASED, SWISS COHORT

Authors

Vonlanthen, Gaël; Marques-Vidal, Pedro

VERSION 1 - REVIEW

**Reviewer**	**1**
**Name**	**Barbosa, Ana**
**Affiliation**	**Universidade do Porto Instituto de Saúde Pública**
**Date**	**19-Sep-2023**
**COI**	**None declared.**

I read with much interest the manuscript "No effect of physical activity in the management of diabetes".

The topic is not new and does not bring novelty to the field.

Furthermore, there are major concerns regarding the conclusions of this paper that arise from a misinterpretation of the study analysis (cross-sectional).

First, the title does not reflect the study results. I would expect to read that there are no differences between PA levels in patients with controlled or not-controlled diabetes.

Second, the interpretation of PA levels should be made with caution. When we perform a cross-sectional analysis, there is no time sequence between exposure and the outcome, therefore I cannot assume that there will be differences in PA levels because this depends on the time of evaluation.

Third, the authors assume "sedentary" as a PA intensity when it is already well-known that sedentary behavior is and independent factor.

Lastly, in the analysis, the authors do not follow PA recommendations from international organizations for this population (e.g. WHO), by aggregating the cut-off of moderate and vigorous exercise intensity, which is not correct.

I recommend the authors look deeply into the concepts and analysis and reformulate it since there is potential to publish the data from this cohort.

VERSION 1 - AUTHOR RESPONSE

Please see the corresponding file

Reviewer: 1

Mrs. Ana Barbosa, Universidade do Porto Instituto de Saúde Pública

Comments to the Author:

I read with much interest the manuscript "No effect of physical activity in the management of diabetes".

1. The topic is not new and does not bring novelty to the field.

   Our answer: we agree that the topic is not new, but our study provides information on objectively assessed physical activity, which is not common, and was conducted in a real-life setting, not in an experimental one. Further, it provides information to Swiss practitioners, which is important at the local level, and serves as further reference to indicate that adequate physical activity levels are little implemented among patients with T2DM.

2. Furthermore, there are major concerns regarding the conclusions of this paper that arise from a misinterpretation of the study analysis (cross-sectional).

   Our answer: we strongly disagree. Cross-sectional studies allow assessing associations between conditions and draw important information regarding the quality of management of noncommunicable diseases, including cardiovascular risk factors. We challenge the reviewer to refute the results of the Portuguese national health survey regarding control of diabetes 10.1016/j.diabres.2018.03.052, based on the fact that it is cross-sectional. Further, our conclusion is based on the finding of no significant differences in physical activity levels between participants with T2DM who have their condition controlled and uncontrolled. We do not conclude that physical activity is useless regarding management of T2DM.

   We changed the conclusion in the abstract to.

   "no differences in PA levels were found between participants with controlled and uncontrolled T2DM"

3. First, the title does not reflect the study results. I would expect to read that there are no differences between PA levels in patients with controlled or not-controlled diabetes.

   Our answer: as per the editor's recommendations, the title has been changed to

   "Association between physical activity and diabetes control: multiple cross-sectional studies and a prospective study in a population-based, Swiss cohort"

4. Second, the interpretation of PA levels should be made with caution. When we perform a cross-sectional analysis, there is no time sequence between exposure and the outcome, therefore I cannot assume that there will be differences in PA levels because this depends on the time of evaluation.

   Our answer: we disagree. Patients with T2DM are supposed to exert physical activity on a regular basis, as per WHO or ESC/EASD recommendations. Our questionnaire assessed physical activity for the last 4 weeks and the accelerometer assessed physical activity for a complete week. Hence, it would be expected that participants with controlled T2DM would present higher physical activity levels irrespective of the time of assessment. This higher compliance would have led to a better control of their diabetic status, which was not found in our study. It would be the same as checking if participants taking statins are adequately controlled: you don't need a time lag to assess this condition, you just check if lipid levels among participants taking statins are lower than those of participants not taking statins.

5. Third, the authors assume "sedentary" as a PA intensity when it is already well-known that sedentary behavior is and independent factor.

   Our answer: we agree that sedentary behaviour is not part of physical activity. Still, in a previous publication, we have shown that sedentary status is associated with increased risk of diabetes [1]. Hence, we decided to include it in the analyses. We added the following statement to the methodology, self-reported physical activity:

   "We chose to include sedentary behaviour in the analysis as we have previously shown that it is associated with an increased risk of developing T2DM [1]."

   We also changed the titles of the chapters to

   "Sedentary behaviour and physical activity levels according to…"

6. Lastly, in the analysis, the authors do not follow PA recommendations from international organizations for this population (e.g. WHO), by aggregating the cut-off of moderate and vigorous exercise intensity, which is not correct.

   Our answer: We used the guidelines from the ESC/EASD regarding physical activity, which explicitly state that a combination of MPA+VPA should be used "Moderate-to-vigorous physical activity, notably a combination of aerobic and resistance exercise, for ≥150 min/week is recommended for the prevention and control of DM, unless contraindicated, such as when there are severe comorbidities or a limited life expectancy » [2], key message 6.1, page 17. We changed the statement in the methods to

   "Participants were considered as complying with the recommendations if the weekly amount of MPA and VPA exceeded 150 minutes, as per as per European Society of Cardiology/ European Association for the Study of Diabetes (ESC/EASD) guidelines [2]."

7. I recommend the authors look deeply into the concepts and analysis and reformulate it since there is potential to publish the data from this cohort.

   Our answer: we made the changes considered as necessary.

References

1. Liu K, Marques-Vidal P. Sleep well, but be active. Effect of sleep and sedentariness on incidence of diabetes. *Prim Care Diabetes* 2023;17(5):454-59. doi: 10.1016/j.pcd.2023.08.002 [published Online First: 2023/08/21]
2. Cosentino F, Grant PJ, Aboyans V, et al. 2019 ESC Guidelines on diabetes, pre-diabetes, and cardiovascular diseases developed in collaboration with the EASD. *Eur Heart J* 2020;41(2):255-323. doi: 10.1093/eurheartj/ehz486 [published Online First: 2019/09/10]

VERSION 2 - REVIEW

**Reviewer**	**2**
**Name**	**Mboya, Innocent**
**Affiliation**	**Lund University, Department of Translational Medicine**
**Date**	**18-Jun-2024**
**COI**	**None**

The authors have done their best to address the editorial and reviewer comments.

**Reviewer**	**3**
**Name**	**Shamsutdinova, Diana**

Affiliation **King's College London, Biostatistics and Health Informatics**

Date **13-Aug-2024**

COI **None**

The study "ASSOCIATION BETWEEN PHYSICAL ACTIVITY AND DIABETES CONTROL: MULTIPLE CROSS-SECTIONAL STUDIES AND A PROSPECTIVE STUDY IN A POPULATION-BASED, SWISS COHORT" utilises the CoLaus study data of Lausanne, Switzerland residents, and investigates the levels of physical activity (PA) in people taking diabetes medication. The main focus is on testing whether the PA measures differ among those with controlled and uncontrolled type 2 diabetes. Controlled T2DM is defined by the blood glucose level being below the diabetes cut off levels. The study data provided both self-reported and wearable device measured PA, at three different time points. Analytical sample consists of around 200 people varied across time points. The authors found no statistically significant differences in PA between the participants with controlled or uncontrolled T2DM.

Apart from those mentioned in the paper, recent report by Low et al (2023) seems very similar to the one presented here (https://fis-db.dshs-koeln.de/ws/portalfiles/portal/17499871/BOA_Paris_2023_Web.pdf#page=917), using the same wearable device and reporting no association between the HBA1C and PA.

I appreciate the topic of the study, as the relationship between the PA and diabetes control is relevant for clinical practice as well as for those living with diabetes and can guide the decisions around the T2DM management. It was also informative to read about the differences in the self-reported and device-collected PA measurements in this study. However, there are several concerns.

First, the sample size is rather small (50-120 participants per group), while the regression is adjusted for quite a few controlled variables (sex, age, BMI (2 categories apart from the baseline), smoking status (2 categories apart from the baseline), educational level (2 categories apart from the baseline)), which further dilutes statistical power. In this case, the inconclusive results could be due to not enough data supplied to detect the differences. Statistically, not categorising the HBA1C or FLG levels (into controlled and uncontrolled) and treating them as continuous variables could be a better option with a higher statistical power (with the idea of regressing the HBA1C on PA levels while controlling for other covariates to address the study question). Reducing number of categories and using continuous instead of categorical wherever possible (e.g., for BMI) can further help to recover the power. I am also left wondered if the PA levels were measured as number of people regularly practicing some level of exercise, would the results be the same? (Here, the average daily times are be compared.)

Second, there is a methodological consideration regarding the inclusion criteria of the analytical sample. The sample is defined as "Participants were considered as presenting with

treated diabetes if they reported taking any antidiabetic drug. ", which seems vague (e.g. which drugs?) and can be clarified further. Does this mean that participants did not have to be diagnosed with T2DM? There could there be a potential bias to participants with other diabetes types than type 2, people with pre-diabetes, and other disorders for which diabetes drugs could be prescribed. It may be reasonable to assume that most of such people do have T2DM, but such an assumption should be spelled out.

Third, the main independent variable, PA activity, seem to be quite different across the 3 reported ways of its measurement, that is, self-reported PA, and PA computed from the wearable device data using the Excel macro and the R package. Authors' thoughts on how that affects results are scattered across the paper, but it may be good to discuss it in more depth in the discussion.

Finally, there is little to no discussion on the implications of these results for the practitioners or patients (is there any; which, if yes; if not, why; what should be researched further). For example, it may be good to acknowledge that cross-sectional design cannot address the question on how effective PA interventions could be but might highlight the fact that people seem to exhibit similar PA behaviour irrespective of their glucose levels in T2DM. More ideas on how the presented results could be explained (apart from those mention in lines 307-318), perhaps, reflecting on how in practice the doctors recommend PA (as the author is a clinician).

Other minor comments that were noted:

Abstract, lines 45-50: not clear is the 90% and 20% of participants with moderate and vigorous PA in the objective assessment were 90% in the 1st and 20% in the 2nd questionnaire, so 90% for moderate and 20% for vigorous PA, please clarify.

(here: "Using subjective data, over 90% (first survey) and 75% (second survey) of participants reported moderate and vigorous PA >150 min/week. After multivariable adjustment, no differences were found regarding all types of self-reported PA levels between controlled and uncontrolled participants. Objective assessment of PA led to considerable differences according to the software used: 90% and 20% of participants with moderate and vigorous PA >150 min/week, respectively. ")

Methods, lines 132-153: Some clarification is needed for the two analyses of the accelerometery data are described: is the first analysis defining the valid days and the second the levels of PA? or are those two alternative methods to measure PA? Please add a sentence or two on how these analyses are connected.

Methods, line 144: "A valid day was defined as ≥10 h (i.e., 600 min-epoch) and ≥8 h (i.e., 480 min-epoch) of diurnal wear-time on weekdays and weekend days, respectively. " - I read "diurnal" as "daily" which assumes averaging, but the authors seemed to mean just the days with more than 10h of wearing the device (continuous?), maybe less confusing to omit this word or re-phrase.

Throughout and lines 166-168: it would be better to be more specific, and use the term "type 2 diabetes"/T2DM; particularly important for eligibility and exclusion criteria section (166-168)

Diabetes assessment 154-158: please add a sentence how the two conditions (FPG < 7 and HBA1C <6.5%) were used to define controlled diabetes (both/ either / whichever available etc)

Methods: Could the authors the details of the statistical analysis conducted with respect to the main question on differences in PA between the controlled and not controlled T2DM. It is the mean daily average of e.g. light PA (in minutes) is compared using linear regressions? (e.g. as opposed to analysing he differences in the number of people reaching a specific level of PA). Which were the binary outcomes for which multivariable logistic regressions were employed? Any analysis of the temporal trends? Are those mostly the same people in all the surveys throughout the time or different?

Statistical analysis:

Table 1, 2: Not clear, what are the +/- (later I see that it is indicated in the supplementary materials). For example, "Vigorous activity 32+/-8 min/day", would that be 8 min of standard deviation across the valid days? It could be that people had different number of eligible days, in which the estimates for the standard deviation are weak.

Discussion, lines 302-306: This seems like an important unresolved question: "According to the GENEActiv macro, almost all participants treated for T2DM were compliant with the current PA recommendations, while according to the R-package GGIR this percentage was less than 25%. These differences between analytical methods have been reported previously [24] and raise the importance of standardization of PA accelerometery measurements [25]" Could the authors state their opinion on this, and which analysis they used as the main one (seems the Excel macro?) and some explanation on why.

326-327 "a population-based sample was used" - this seems a strong statement for the sample size

---

VERSION 2 - AUTHOR RESPONSE

Dr. Diana Shamsutdinova, King's College London

Comments to the Author:

1. The study "ASSOCIATION BETWEEN PHYSICAL ACTIVITY AND DIABETES CONTROL: MULTIPLE CROSS-SECTIONAL STUDIES AND A PROSPECTIVE STUDY IN A POPULATION-BASED, SWISS COHORT" utilises the CoLaus study data of Lausanne, Switzerland residents, and investigates the levels of physical activity (PA) in people taking diabetes medication.

The main focus is on testing whether the PA measures differ among those with controlled and uncontrolled type 2 diabetes. Controlled T2DM is defined by the blood glucose level being below the diabetes cut off levels. The study data provided both self-reported and wearable device measured PA, at three different time points. Analytical sample consists of around 200 people varied across time points. The authors found no statistically significant differences in PA between the participants with controlled or uncontrolled T2DM.

2. Apart from those mentioned in the paper, recent report by Low et al (2023) seems very similar to the one presented here (https://fis-db.dshs-koeln.de/ws/portalfiles/portal/17499871/BOA_Paris_2023_Web.pdf#page=917), using the same wearable device and reporting no association between the HBA1C and PA.

   Our answer: we were not aware of this abstract. We thank the reviewer for indicating it and it is now referenced (reference number 34).

I appreciate the topic of the study, as the relationship between the PA and diabetes control is relevant for clinical practice as well as for those living with diabetes and can guide the decisions around the T2DM management. It was also informative to read about the differences in the self-reported and device-collected PA measurements in this study. However, there are several concerns.

1. First, the sample size is rather small (50-120 participants per group), while the regression is adjusted for quite a few controlled variables (sex, age, BMI (2 categories apart from the baseline), smoking status (2 categories apart from the baseline), educational level (2 categories apart from the baseline)), which further dilutes statistical power. In this case, the inconclusive results could be due to not enough data supplied to detect the differences. Statistically, not categorising the HBA1C or FLG levels (into controlled and uncontrolled) and treating them as continuous variables could be a better option with a higher statistical power (with the idea of regressing the HBA1C on PA levels while controlling for other covariates to address the study question). Reducing number of categories and using continuous instead of categorical wherever possible (e.g., for BMI) can further help to recover the power. I am also left wondered if the PA levels were measured as number of people regularly practicing some level of exercise, would the results be the same? (Here, the average daily times are be compared.)

   Our answer: we reanalysed the data using FPG and HbA1c as continuous variables using linear regression and provide the results as standardized beta coefficients to facilitate comparisons. Results are in supplementary tables 6 to 8 for FPG and supplementary tables 12 and 13 for Hb1c. Besides a significant negative association between light physical activity and FPG and Hba1c levels in the second follow-up (which was not replicated in the third follow-up), no other association was found. We added the following statement in the methods, statistical analysis:

   "A sensitivity analysis was conducted using multivariable linear regression adjusting for the same covariates to assess the association between PA and fasting plasma glucose or glycated hemoglobin. Results were expressed as standardized beta coefficients."

We added the following statements in the results, end of chapter Sedentary behaviour and physical activity levels according to diabetes control as per fasting plasma glucose

"The results of the sensitivity analysis using multivariable linear regression are provided in supplementary tables 6 to 8. Besides a significant negative association between light physical activity and glucose levels in the second follow-up for PA assessed by the MACRO procedure, which was not confirmed in the third follow-up, no other association between PA levels and glucose levels was found."

And at the end of the chapter Sedentary behaviour and physical activity levels according to diabetes control as per glycated haemoglobin:

"The results of the sensitivity analysis using multivariable linear regression are provided in supplementary tables 12 and 13. No significant association between PA levels and glycated hemoglobin was found."

2. Second, there is a methodological consideration regarding the inclusion criteria of the analytical sample. The sample is defined as "Participants were considered as presenting with treated diabetes if they reported taking any antidiabetic drug. ", which seems vague (e.g. which drugs?) and can be clarified further. Does this mean that participants did not have to be diagnosed with T2DM? There could there be a potential bias to participants with other diabetes types than type 2, people with pre-diabetes, and other disorders for which diabetes drugs could be prescribed. It may be reasonable to assume that most of such people do have T2DM, but such an assumption should be spelled out.

   Our answer: in our study, participants were asked "Est-ce qu'on vous a dit que vous aviez du diabète ? » (Have you been told you have diabetes?) and if the answer was positive, they had to answer the question "Si oui, est-ce que vous prenez des médicaments (y compris insuline) pour traiter le diabète ? » If yes, are you taking any medication (including insulin) to treat diabetes? We believe that this last question indicates that we are focusing on T2DM, not on other types of diabetes. We also checked the drugs that the participants reported taking and they matched with their statements. Prediabetes is not a condition for prescribing antidiabetic drugs in Switzerland, and this prescription would have been rejected (i.e., not reimbursed) by the health insurances. We added the following text in the methods, diabetes assessment:

   "Participants were asked whether they had been told they had diabetes and, if the answer was positive, if they were taking any medication (including insulin) to treat their diabetes. "

3. Third, the main independent variable, PA activity, seem to be quite different across the 3 reported ways of its measurement, that is, self-reported PA, and PA computed from the wearable device data using the Excel macro and the R package. Authors' thoughts on how that affects results are scattered across the paper, but it may be good to discuss it in more depth in the discussion.

Our answer: we now provide a short paragraph focusing on the issues of PA assessment:

"PA levels differed considerably according to the methodology used. The differences between reported and objectively assessed PA are known [1], and the differences in PA levels according to the software used to process the accelerometry data have also been detected previously [2]. Overall, our results indicate that the method to assess PA might considerably impact the associations between PA and cardiometabolic risk factors. Hence, care should be taken when comparing findings from studies that used different software to assess PA."

4. Finally, there is little to no discussion on the implications of these results for the practitioners or patients (is there any; which, if yes; if not, why; what should be researched further). For example, it may be good to acknowledge that cross-sectional design cannot address the question on how effective PA interventions could be but might highlight the fact that people seem to exhibit similar PA behaviour irrespective of their glucose levels in T2DM. More ideas on how the presented results could be explained (apart from those mention in lines 307-318), perhaps, reflecting on how in practice the doctors recommend PA (as the author is a clinician).

Our answer: we now provide a statement regarding the implications for clinical practice.

"Overall, our results suggest that people with diabetes exhibit the same PA behaviour irrespective of their FPG or HbA1c levels. As PA is part of the management of T2DM [3], more emphasis should be put by clinicians to motivate their patients to be more active, different types of PA being effective [4]. Still, doctors might not have either the time or the knowledge [5] to adequately advise their patients regarding PA. Hence, postgraduate training regarding PA prescription is advised [6]."

We also added a short statement in the limitations chapter:

"Third, the cross-sectional design of this study cannot address the question whether effective PA levels can efficiently help manage diabetes. Still, our results are similar to those reported elsewhere [7], and suggest that PA levels should be implemented among people with diabetes."

References

1. Verhoog S, Gubelmann C, Guessous I, et al. Comparison of the Physical Activity Frequency Questionnaire (PAFQ) with accelerometry in a middle-aged and elderly population: The CoLaus study. *Maturitas* 2019;129:68-75. doi: 10.1016/j.maturitas.2019.08.004

2. Verhoog S, Gubelmann C, Bano A, et al. Comparison of different software for processing physical activity measurements with accelerometry. *Sci Rep* 2023;13(1):2879. doi: 10.1038/s41598-023-29872-7

3. Cosentino F, Grant PJ, Aboyans V, et al. 2019 ESC Guidelines on diabetes, pre-diabetes, and cardiovascular diseases developed in collaboration with the EASD. *Eur Heart J* 2020;41(2):255-323. doi: 10.1093/eurheartj/ehz486 [published Online First: 2019/09/10]

4. Pan B, Ge L, Xun Y-Q, et al. Exercise training modalities in patients with type 2 diabetes mellitus: a systematic review and network meta-analysis. *IJBNPA* 2018;15(1):72. doi: 10.1186/s12966-018-0703-3

5. O'Brien MW, Shields CA, Oh PI, et al. Health care provider confidence and exercise prescription practices of Exercise is Medicine Canada workshop attendees. *Appl Physiol Nutr Metab* 2017;42(4):384-90. doi: 10.1139/apnm-2016-0413 [published Online First: 2017/02/09]

6. Windt J, Windt A, Davis J, et al. Can a 3-hour educational workshop and the provision of practical tools encourage family physicians to prescribe physical activity as medicine? A pre-post study. *BMJ open* 2015;5(7):e007920. doi: 10.1136/bmjopen-2015-007920 [published Online First: 2015/07/05]

7. Low J, Hesketh K, Little J, et al. Physical activity levels and cardiometabolic markers in indviduals with newly diagnosed type 2 diabetes: a baseline analysis of the MOTIVATE T2D randomized controlled trial. In: Guilhem G, Rabita G, Brocherie F, et al., eds. 28th Annual Congress of the European College of Sports Science. Paris, France, 2023.
